# CTGF knockdown in Vero cells reduces autophagy and adhesion and promotes short-term suspension adaptation

**DOI:** 10.3389/fbioe.2026.1777187

**Published:** 2026-03-17

**Authors:** Runsheng Peng, Renhou Jia, Rong Huang, Muzi Li, Xiaoyun Li, Manlin Zhou, Jiamin Wang, Zilin Qiao, Na Sun

**Affiliations:** 1 Ministry of Education Engineering Research Centre for Key Technology and Industrialisation of Cell-Based Vaccines, Northwest Minzu University, Lanzhou, Gansu, China; 2 Gansu Tech Innovation Center of Animal Cell, Northwest Minzu University, Lanzhou, Gansu, China; 3 Key Laboratory of Biotechnology and Bioengineering of State Ethnic Affairs Commission, Northwest Minzu University, Lanzhou, Gansu, China; 4 College of Life Sciences and Engineering, Northwest Minzu University, Lanzhou, Gansu, China; 5 Gansu Provincial Bioengineering Materials Engineering Research Center, Lanzhou, Gansu, China

**Keywords:** CTGF, Vero cells, autophagy, suspension adaptation, adhesion, virus replication

## Abstract

**Introduction:**

Vero cells are extensively used in viral vaccine production, but their adaptation to serum-free suspension culture is hindered by excessive autophagy and strong anchorage dependence.

**Methods:**

In this study, we identified Connective Tissue Growth Factor (CTGF/CCN2) as significantly upregulated under starvation—an inducer of autophagy—via RNA-seq screening. A stable CTGF knockdown Vero cell line (knockdown efficiency >50%) was established using lentiviral shRNA.

**Results:**

Functional characterization demonstrated that CTGF depletion concurrently attenuated autophagic flux (evidenced by reduced LC3-II/I ratio and lysosomal activity) and impaired cell adhesion (with adhesion rates decreased by 50%–60% on extracellular matrix proteins), while maintaining normal cell proliferation.

**Discussion:**

Our findings reveal a new role for CTGF in regulating the environmental adaptation of Vero cells by coordinating autophagy and adhesion functions. The engineered cell line provides a novel strategy to overcome suspension adaptation bottlenecks, offering significant potential for improving vaccine production scalability.

## 1 Introduction

The Vero cell line, derived from African green monkey kidney epithelial cells (Ammerman et al., 2008), is a pivotal cellular substrate for the production of human viral vaccines due to its susceptibility to a wide range of viruses, absence of interferon response (Rhim et al., 1969), and non-tumorigenic properties (Andreani et al., 2017). To meet the growing global demand for vaccines, such as polio and rabies vaccines, the biopharmaceutical industry is increasingly transitioning from traditional adherent culture systems to scalable serum-free suspension processes (Rourou et al., 2007). However, the anchorage-dependent nature of Vero cells and their propensity to undergo excessive autophagy under suspension conditions pose significant challenges to achieving high-density, stable growth in bioreactors.

As we know, Genetic engineering has emerged as a powerful tool for developing engineered cell lines with tailored functions. Efforts to adapt Vero cells to suspension culture have encountered persistent challenges. Early studies by Litwin (1992) achieved limited suspension growth in serum-free medium but observed cell aggregation (Litwin, 1992). Subsequently, building on previous adaptation work, Paillet et al. successfully cultured Vero cells in bioreactors using single-cell batch and perfusion modes, though this culture mode was not sustained (Paillet et al., 2009). Recent adaptations using proprietary media by Rourou, Shen, and Logan improved cell survival but revealed persistent issues including prolonged doubling times and pronounced autophagy/mitophagy (Rourou et al., 2019; Shen et al., 2019; Logan et al., 2022). Severe mitophagy and autophagy have also been consistently observed in previous studies (Li et al., 2025). These obstacles reveal the need to regulate autophagy and adhesion for effective suspension adaptation. Thus, effective regulation of autophagy is a key breakthrough for enhancing the performance of Vero cell suspension cultures.

Genetic engineering offers a promising avenue to overcome these limitations. In 2009, Shiloach et al. enabled full suspension growth in MDCK cells by overexpressing the adhesion-modifying gene Siat7e (Chu et al., 2009). Similarly, Xu et al. generated TMPRSS2- and MSPL-expressing Vero lines to enhance PEDV propagation (Wang et al., 2020). These successes demonstrate the potential of targeted genetic interventions to decouple adhesion dependence and balance autophagy. The Cellular Communication Network Factor (CCN) family comprises six secreted cysteine-rich matricellular proteins. Connective Tissue Growth Factor (CTGF), the second member of the CCN family, was first discovered in Human Umbilical Vein Endothelial Cells (HUVECs) (Bradham et al., 1991). Due to its unique structure, CTGF acts as a multifunctional modulator, binding to various ligands and receptors (Jun and Lau, 2011). Under normal physiological conditions, CTGF is involved in embryonic development, chondrogenesis, wound healing, and tissue repair (Ramazani et al., 2018). Studies have shown that CTGF promotes cancer cell proliferation, migration, invasion, metastasis, and Epithelial-Mesenchymal Transition (EMT), playing a critical role in tumorigenesis and progression (Kular et al., 2011; Chen et al., 2007; Kim et al., 2020). Based on this, we hypothesized that knocking down CTGF expression could effectively reduce autophagy levels, reduce cell adhesiveness, and thereby enhance suspension culture adaptability.

This study primarily utilized Earle’s Balanced Salt Solution (EBSS) to induce autophagy in Vero cells. RNA sequencing (RNA-seq) to screen for autophagy-related genes, followed by genetic engineering techniques to develop cell lines with improved suspension adaptability and balanced autophagy. We identified Connective Tissue Growth Factor (CTGF/CCN2) as a key gene upregulated during starvation-induced autophagy in adherent Vero cells. Based on its established roles in modulating autophagy and cell adhesion, we hypothesized that CTGF knockdown could facilitate suspension adaptation. Using lentivirus-mediated shRNA, we generated a stable CTGF-knockdown Vero cell line and evaluated its autophagy activity, adhesion capacity, migration, and proliferation, providing a novel engineered cell resource for scalable vaccine production.

## 2 Materials and methods

### 2.1 Materials

#### 2.1.1 Cells and culture media

Adherent Vero cells were purchased from the American Type Culture Collection (ATCC). M199 medium and 0.25% trypsin were obtained from Lanzhou Bailing Biotechnology Co., Ltd. Fetal bovine serum (FBS) was purchased from Lanzhou Minhai Bioengineering Co., Ltd.

#### 2.1.2 Main reagents and antibodies

TRIzol, Evo M-MLV Reverse Transcription Premix Kit, and qRT-PCR Kit were acquired from Hunan Accura Biology Co., Ltd. BCA Protein Quantification Kit and EdU Cell Proliferation Kit with Alexa Fluor 555 were obtained from Dalian Meilun Biotechnology Co., Ltd. RIPA lysis buffer, protease inhibitor, 5× SDS-PAGE protein loading buffer, and DAPI (4′,6-diamidino-2-phenylindole dihydrochloride) staining mounting medium were purchased from Beijing Solarbio Science and Technology Co., Ltd. Rabbit-derived LC3, rabbit-derived P62, mouse-derived β-actin, and mouse-derived GAPDH antibodies were obtained from Hangzhou Hua’an Biotechnology Co., Ltd. Rabbit-derived Bax and Bcl-2 antibodies were purchased from Abmart Medical Technology Co., Ltd. Horseradish peroxidase (HRP)-labeled goat anti-rabbit IgG and HRP-labeled goat anti-mouse IgG were acquired from Beijing Zhongshan Jinqiao Biotechnology Co., Ltd. TRITC Phalloidin was obtained from Yeasen Biotechnology Co., Ltd. Hoechst 33,342 was purchased from AAT Bioquest (Sunnyvale, CA, USA).

### 2.2 Methods

#### 2.2.1 RNA-seq sequencing of Vero cells under starvation stress

Total RNA from triplicate samples of non-starved and EBSS-induced starved Vero cells was extracted using TRIzol reagent. After quality assessment, poly(A)+ mRNA was enriched for library construction. The libraries were sequenced on the DNBSEQ platform. Following sequencing, raw reads were processed to obtain clean data, which were then aligned to the reference genome for novel transcript prediction and expression quantification. Differential expression analysis was performed using the DESeq2 package in R. Genes with nominal p-value <0.05 were considered as nominally differentially expressed. For identification of significantly differentially expressed genes (DEGs), we applied more stringent thresholds: |Fold Change| > 1.5 and FDR-adjusted p-value (q-value) <0.05. This identified 80 significantly upregulated and five significantly downregulated genes. Finally, KEGG pathway enrichment analysis of the DEGs was conducted, with an FDR q-value <0.05 considered statistically significant. The more stringent criteria were used for downstream pathway enrichment analysis to ensure robustness of the findings.

#### 2.2.2 RT-qPCR analysis

Total RNA was extracted from approximately 3 × 10^6^ harvested Vero cells using TRIzol reagent, and its concentration and purity (OD260/280: 1.8–2.0) were measured. Subsequently, 1 μg of total RNA was used for genomic DNA removal and reverse transcription to synthesize cDNA. Quantitative real-time PCR (qRT-PCR) was performed using the SYBR Green method with gene-specific primers (see Table 1 for sequences) and GAPDH as the internal reference. The thermal cycling conditions consisted of an initial denaturation at 95 °C for 30 s, followed by 40 cycles of 95 °C for 5 s and 60 °C for 30 s, concluding with a melt curve analysis. The relative expression levels of target mRNAs were calculated using the 2^−ΔΔCt^ method.

#### 2.2.3 Preparation and transfection of sh-CTGF lentivirus

To establish stable CTGF-knockdown Vero cell lines, lentiviral vectors were constructed by cloning designed shRNA sequences targeting the CTGF gene (NCBI:https://www.ncbi.nlm.nih.gov/) into the GV493 backbone. The recombinant plasmids were verified by sequencing and subsequently co-transfected with packaging plasmids (Help 1.0/2.0) into 293T cells for virus production. The harvested lentiviral particles were concentrated by ultracentrifugation to a titer of ≥1 × 10^8^ TU/mL. For transduction, Vero cells were infected with the virus at a multiplicity of infection (MOI) of 60. Stable polyclonal cell lines (shCTGF (49), shCTGF (50), and a control shCTGF con) were then selected under 4 μg/mL puromycin treatment for 10 continuous passages. The knockdown efficiency was ultimately validated at the protein level by Western blot analysis.

#### 2.2.4 Western blot (WB) analysis

Total protein was extracted from cells at 70%–80% confluence using RIPA lysis buffer combined with repeated freeze-thaw cycles. Protein concentration was determined with a BCA assay, and samples were denatured with 5× SDS loading buffer by boiling at 100 °C for 10 min. The proteins were then resolved on 10%–12% SDS-PAGE gels (80 V through stacking gel, 120 V through separating gel) and subsequently transferred onto PVDF membranes at 75 V for 90 min. Finally, the membranes were blocked with 5% skim milk in TBST at room temperature for 2.5 h prior to immunoblotting.

#### 2.2.5 Immunofluorescence assay

For immunofluorescence staining, cells grown on coverslips were fixed with 4% paraformaldehyde for 15 min, permeabilized with 0.5% Triton X-100 for 30 min, and blocked with 10% goat serum. Subsequently, the samples were incubated overnight at 4 °C with primary antibody, followed by a 1-h incubation at room temperature with fluorescent secondary antibody protected from light. Cell nuclei were counterstained with DAPI.Finally, the coverslips were mounted with an anti-fade mounting medium.Fluorescence intensity was examined using a fluorescence microscope (Axiovert 5, ZEISS) and quantified with ImageJ software.

#### 2.2.6 Lysosomal acidity and cathepsin B activity assay

Cells grown to 70%–80% confluence in 35 mm glass-bottom dishes were used. For lysosomal acidity assessment, cells were incubated with 20 nM LysoBrite Red, while cathepsin B activity was detected using 1 nM Magic Red CTSB substrate, both in pre-warmed EBSS at 37 °C for 30 min according to the respective protocols. After washing, all samples were counterstained with Hoechst 33,342 at 37 °C for 15 min. Following final washes, serum-free M199 medium was added, and live-cell imaging was performed on a confocal microscope (ZEISS, LSM900). Fluorescence intensity was quantified using ImageJ software.

#### 2.2.7 Cell adhesion assay

Cell adhesion was assessed by DAPI staining of adherent cells. Briefly, 96-well plates were coated with 10 μg/mL fibronectin (FN), collagen I (Col I), or laminin (LN) at 37 °C for 12 h, washed with PBS, and blocked with 1% BSA in serum-free DMEM for 1 h. Cells at 70%–80% confluence were trypsinized, counted, and seeded into the coated wells at 1 × 10^4^ cells/well. After 30 min incubation at 37 °C, non-adherent cells were removed by washing with PBS. Adherent cells were fixed with 4% paraformaldehyde for 15 min, stained with DAPI for 10 min, and imaged under a fluorescence microscope. The number of adherent cells in randomly selected fields was counted using ImageJ software. Adhesion rate was calculated as: (number of adherent cells in experimental wells/number in control wells) × 100%, where control wells were uncoated. All experiments were performed in triplicate and repeated independently at least three times.

#### 2.2.8 Cell wound healing (scratch) assay

Cells were cultured in 6-well plates until reaching 70%–80% confluence. After washing with PBS, uniform scratches were created in each well using a sterile pipette tip (three scratches per well as replicates), and dislodged cells were removed by washing. Subsequently, 2 mL of M199 medium supplemented with 5% NBS was added to each well. Cell migration into the scratch area was monitored and photographed at 0, 6, 12, 24, 36, and 48 h under a microscope. The wound closure rate was quantified using ImageJ software.

#### 2.2.9 F-actin staining to assess cell adhesion capacity

Cells grown on coverslips to 70%–80% confluence were fixed with 4% paraformaldehyde for 15 min and permeabilized with 0.5% Triton X-100 for 30 min. Subsequently, F-actin was labeled by incubation with 20 nM TRITC-phalloidin in the dark at room temperature for 30 min, and nuclei were counterstained with DAPI. Following final washes, coverslips were mounted with an anti-fade medium. Images were acquired using a fluorescence microscope (ZEISS,LSM900), and fluorescence intensity was quantified with ImageJ software.

#### 2.2.10 Cell growth curve assay

Cells in the logarithmic growth phase (70%–80% confluence) were trypsinized to prepare a single-cell suspension, which was then counted and seeded at equal density into 24-well plates. Cell numbers from triplicate wells were serially monitored daily for seven consecutive days. A growth curve was subsequently plotted with culture time as the X-axis and cell density as the Y-axis.

#### 2.2.11 EdU assay for cell proliferation

Cells cultured in 6-well plates at 50%–60% confluence were labeled with 10 μM EdU for 24 h. Following labeling, cells were fixed with 4% paraformaldehyde, permeabilized with 0.5% Triton X-100, and subjected to a Click reaction for EdU detection according to the manufacturer’s instructions. Nuclei were then counterstained with Hoechst 33,342. Fluorescence images (EdU and Hoechst) were acquired using a fluorescence microscope (ZEISS, LSM900), and the percentage of EdU-positive cells was quantified with ImageJ software.

#### 2.2.12 Flow cytometry for apoptosis assay

Cells cultured to 70%–80% confluence were harvested, washed, and resuspended in binding buffer. For apoptosis analysis, cells were stained with Annexin V-FITC at room temperature in the dark for 10 min, followed by propidium iodide (PI) staining on ice in the dark. Samples were then analyzed immediately using a flow cytometer. Apoptotic populations were distinguished based on Annexin V-FITC (green fluorescence) and PI (red fluorescence) signals.

#### 2.2.13 Transmission electron microscopy

Cells at 70%–80% confluence were starved in EBSS for 2 h, harvested, and fixed sequentially with 2.5% glutaraldehyde and 1% osmium tetroxide. Following fixation, samples were dehydrated through a graded acetone series, infiltrated, and embedded in Epon-812 resin. Ultrathin sections (60–90 nm) were cut using a ultramicrotome (LEICA), stained with uranyl acetate and lead citrate, and examined under a transmission electron microscope (JEOL JEM-1400FLASH). Images were acquired at both low magnification for overview and high magnification to resolve ultrastructural details.

#### 2.2.14 Tissue culture infectious dose 50

The 50% tissue culture infectious dose (TCID50) was calculated using the Reed-Muench method. Vero cells were seeded in 96-well plates and inoculated with 10-fold serial dilutions of the virus. After incubation, the cytopathic effect (CPE) in each well was observed, and the viral titer was determined according to the Reed-Muench formula.

#### 2.2.15 Statistical analysis

All quantitative data are presented as mean ± standard deviation (SD) from at least three independent experiments. Statistical analysis was performed using GraphPad Prism software (version 9.5). Differences between two groups were assessed by an unpaired Student’s t-test, while comparisons among multiple groups were analyzed by one-way analysis of variance (ANOVA). A P value of less than 0.05 was considered statistically significant.

## 3 Results

### 3.1 Vero cells are induced autophagy by EBSS

**FIGURE 1:**
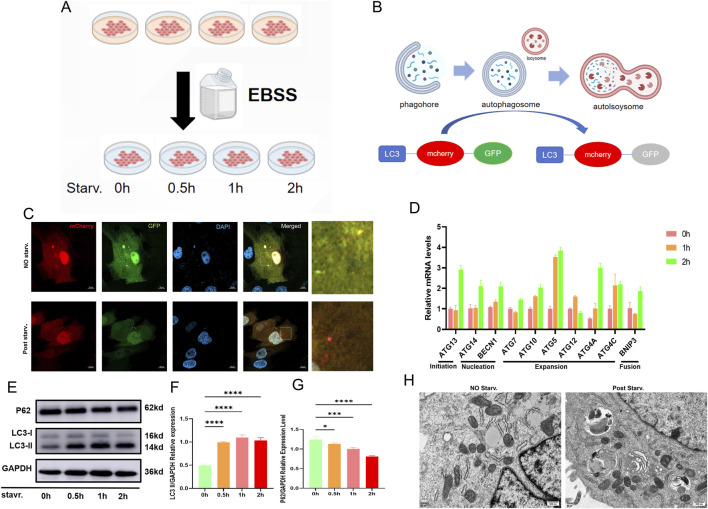
Vero cells are activated for autophagy by EBSS. **(A)** Schematic diagram of the EBSS-induced starvation model in Vero cells. **(B)** LC3 tagged with mCherry and GFP: yellow fluorescence indicates no autophagy (both fluorophores active); red fluorescence indicates autophagy (GFP quenched in acidic conditions, mCherry retained). **(C)** GFP quenching and mCherry retention under starvation induction, indicating autophagy. **(D)** RT-qPCR detection of autophagy gene expression at various stages under starvation. **(E)** Western blot analysis of autophagy marker proteins LC3 and P62 under starvation induction. **(F,G)** Densitometric analysis confirmed the increased conversion of LC3-I to LC3-II and the gradual decrease of p62 in Vero cells post-autophagy (n = 3 per group; *: *p* < 0.05, **: *p* < 0.01, ***: *p* < 0.001, ****: *p* < 0.0001, ns: *p* > 0.05). **(F)** Quantitative analysis of LC3-II. **(G)** Quantitative analysis of P62. **(H)** Under hunger-induced conditions, TEM (Transmission Electron Microscopy) was used to observe the formation of autophagosomes and mitophagy.

Based on previously adapted suspended Vero cells in our laboratory, RNA-seq comparison between suspended and adherent Vero cells revealed significant enrichment of autophagy-related pathways. To facilitate precise quantification and visualization of autophagy, which is technically demanding in suspension, we established a EBSS-induced autophagy model using adherent Vero cells (Figure 1A). Firstly,we adopted a fluorescence reporter that fused LC3,a autophagy maker pretein, with mCherry and GFP to monitor the occurrence of autophagy. When autophagy occurs, mCherry-GFP-LC3 only emitted red fluorescence (mCherrypositive and GFP-negative puncta) (Figure 1B), showing significantly increased autophagic flux after EBSS starvation (Figure 1C). Through RT-qPCR detected upregulated transcripts of autophagy-related genes during autophagy induction (Figure 1D), and Western blot analysis of LC3 and P62 showed a gradual increase in the LC3-II ratio and a gradual decrease in P62 (Figure 1E) (Figure 1F) (Figure 1G), confirming significant autophagy induction. Furthermore, transmission electron microscopy (TEM) revealed that a higher occurrence of autophagy after induction with EBSS compared to the pre-starvation state (Figure 1H). These results suggest that EBSS can induce some autophagy in Vero cells.

### 3.2 Transcriptomic analysis of Vero cells after autophagy by EBSS

**FIGURE 2:**
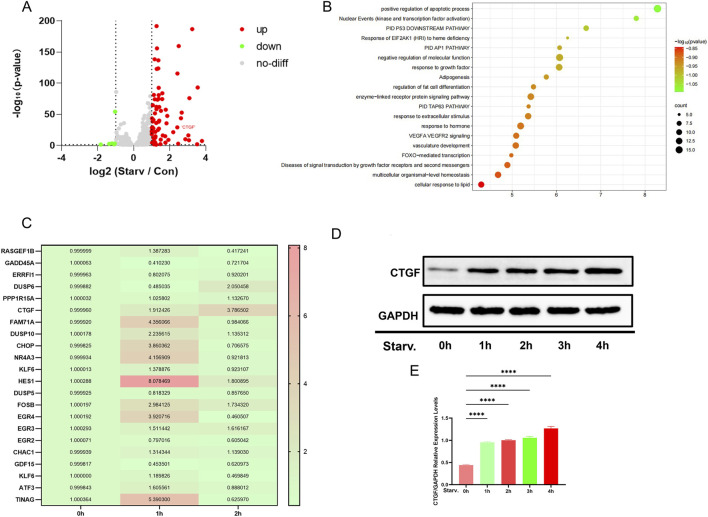
Transcriptomic analysis after starvation. **(A)** Volcano plot of RNA-seq-detected differentially expressed genes (DEGs); Log_2_ (starv/con) < −1 indicates downregulation, Log_2_ (starv/con) > 1 indicates upregulation. **(B)** KEGG pathway enrichment analysis of upregulated DEGs. **(C)** RT-qPCR validation of genes with Log_2_ (starv/con) > 2 under starvation. **(D)** Western blot detection of time-dependent changes in CTGF protein expression under starvation induction. **(E)** Densitometric analysis confirmed the gradual accumulation of CTGF in Vero cells post-autophagy (n = 3 per group; *: *p* < 0.05, **: *p* < 0.01, ***: *p* < 0.001, ****: *p* < 0.0001, ns: *p* > 0.05).

Through RNA-seq was performed after 1 h of EBSS stimulation. Volcano plot analysis identified 1210 DEGs, including 80 significantly upregulated (Log_2_ (starv/con) > 1) and five significantly downregulated (Log_2_ (starv/con) < −1) genes (Figure 2A). Based on KEGG pathway analysis, we found that they can be divided into four functional modules: apoptosis and survival regulation (e.g., positive regulation of apoptotic process, P53 pathway), signal transduction (e.g., AP1 pathway), stress response (e.g., response to growth factor), and metabolic homeostasis (e.g., adipogenesis). Among these modules, the “positive regulation of apoptotic process” pathway shows the highest statistical significance (Figure 2B).Then perform RT-qPCR validation on Log_2_ (starv/con) > 2, it was found that CTGF increases dynamically (Figure 2C), consistent with Western blot results (Figure 2D) (Figure 2E).While CTGF is a well-documented mediator of cell adhesion (Jiang et al., 2013), its potential involvement in autophagy regulation is unknown. our transcriptomic analysis revealed a significant upregulation of CTGF during EBSS-induced autophagy, a context in which its function remains unexplored. This concurrent elevation prompted us to hypothesize that CTGF may serve as a nexus, linking the regulation of autophagy with adhesion. To test this hypothesis and address the dual bottlenecks of excessive autophagy and strong adhesion in Vero cell suspension culture, we targeted CTGF for knockdown, aiming to generate an engineered cell line with enhanced suspension adaptability.

### 3.3 Low expression CTGF in Vero cells had no infect on epithelial-like characteristics

**FIGURE 3:**
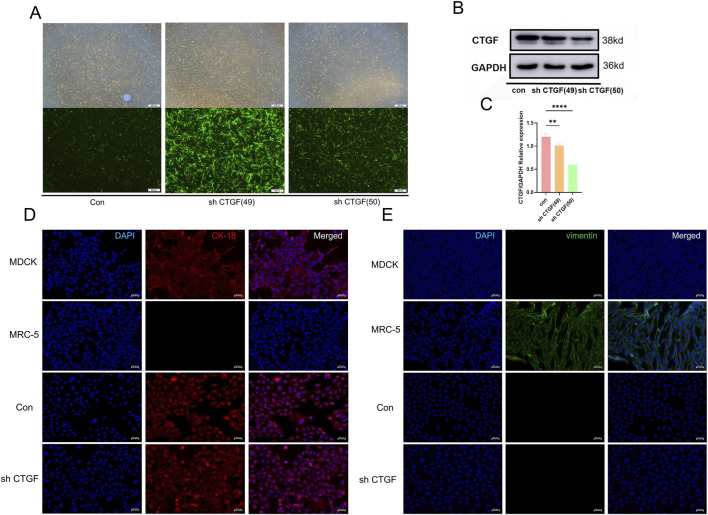
Construction of Vero cells with CTGF knockdown and no change in epithelial-like characteristics. **(A)** Fluorescence intensity of cells after 10 passages of antibiotic selection. **(B)** Western blot detection of CTGF expression levels in cells after 10 passages of selection. **(C)** Densitometric analysis confirmed the significant knockdown effect of CTGF following lentiviral transduction (n = 3 per group; *: *p* < 0.05, **: *p* < 0.01, ***: *p* < 0.001, ****: *p* < 0.0001, ns: *p* > 0.05). **(D)** Immunofluorescence identification of epithelial-like characteristics using Cytokeratin-18 (epithelial marker) and **(E)** Vimentin (fibroblast marker).

Following lentiviral transfection (Supplementary Figure S1), Western blot analysis confirmed a CTGF knockdown efficiency of >50% (Figure 3A) (Figure 3B) (Figure 3C), indicating successful knockdown. Since CTGF is a key marker of Epithelial-Mesenchymal Transition (EMT) (Schober et al., 2002), immunofluorescence for Vimentin (fibroblast-specific) and CK-18 (epithelial-specific) was performed.It was found that Vimentin is not expressed in sh CTGF cells compared to MRC-5 cells (fibroblast cells) (Figure 3D), while ck-18 is normally expressed in sh CTGF cells, consistent with MDCK cells (epithelial cells) (Figure 3E). These results confirm that CTGF knockdown does not alter the epithelial characteristics of Vero cells.

### 3.4 Low expression of CTGF in vero cells reduce autophagy capacity

**FIGURE 4:**
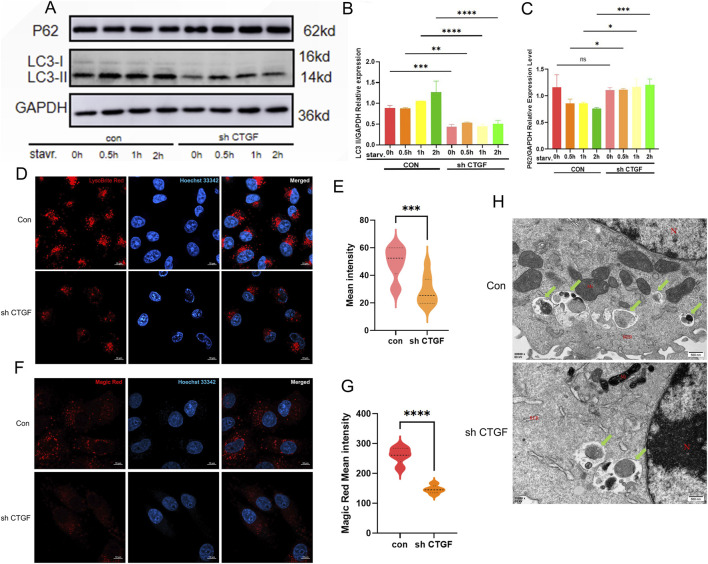
Knock down of CTGF in Vero Cells reduce Autophagy Capacity. **(A)** Western blot analysis of autophagy marker proteins P62 and LC3 after CTGF knockdown (shCTGF). **(B,C)** Densitometric analysis confirmed the decreased conversion of LC3-I to LC3-II and the gradual accumulation of p62 following knockdown. **(B)** Quantitative analysis of LC3-II. **(C)** Quantitative analysis of P62. **(D)** Lysosome formation detected by LysoBrite Red staining after CTGF knockdown (shCTGF) and **(E)** its quantitative results. **(F)** Cathepsin B activity detected by Magic Red staining after CTGF knockdown (shCTGF) and **(G)** its quantitative results. (n = 3 per group; *: *p* < 0.05, **: *p* < 0.01, ***: *p* < 0.001, ****: *p* < 0.0001, ns: *p* > 0.05). **(H)** Autophagy levels in knockdown cells under starvation conditions were observed by TEM. Green arrows indicate the occurrence of autophagy.

Based on Western blot analysis, the LC3-I/II ratio was significantly decreased in shCTGF Vero cells compared to control cells, while P62 expression was upregulated collectively indicating impaired autophagic flux (Figure 4A) (Figure 4B) (Figure 4C). Consistently, LysoBrite Red staining revealed a marked reduction in lysosomal number in shCTGF cells (Figure 4D) (Figure 4E), and Magic Red staining showed decreased cathepsin B activity, reflecting diminished lysosomal function (Figure 4F) (Figure 4G). Furthermore, TEM analysis of starved cells demonstrated a clear reduction in autophagosome formation and mitophagy in the shCTGF group compared to the control (Figure 4H). Together, these results demonstrate that CTGF knockdown suppresses autophagic activity by impairing lysosomal biogenesis and function, thereby blocking autohagic progression.

### 3.5 CTGF knockdown reduces adhesion capacity in Vero cells

**FIGURE 5:**
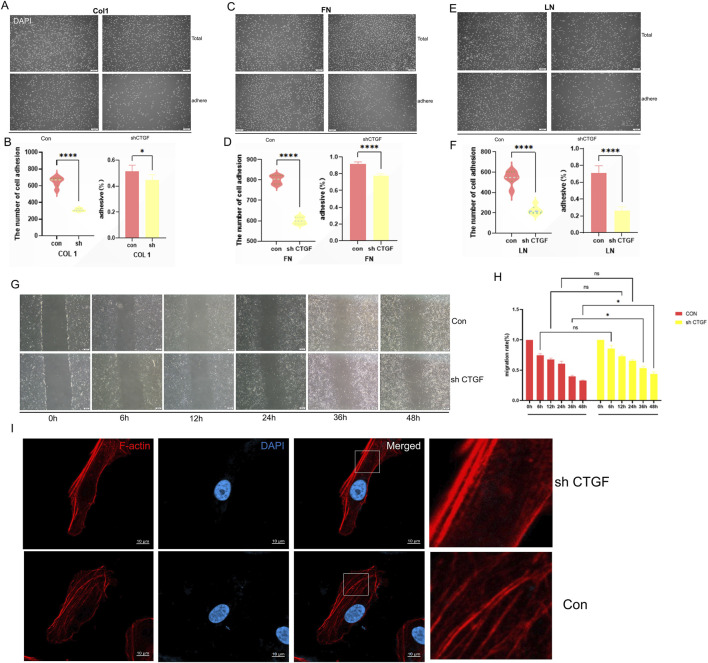
Knock down of CTGF in Vero Cells (sh CTGF)reduce Adhesion, Migration Capacity and stress fiber formation. **(A)** Fluorescence detection of cell adhesion number on Type I collagen matrix. **(B)** Quantitative analysis of the number and rate of adherent cells on Type I collagen matrix. **(C)** Cell adhesion number on fibronectin (FN) matrix. **(D)** Quantitative analysis of the number and rate of adherent cells on fibronectin (FN) matrix. **(E)** Cell adhesion number on laminin (LN) matrix. **(F)** Quantitative analysis of the number and rate of adherent cells on laminin (LN) matrix. **(G)** Cell migration ability assessed by wound healing assay and **(H)** its quantitatively calculated migration rate results (n = 3 per group; *: *p* < 0.05, **: *p* < 0.01, ***: *p* < 0.001, ****: *p* < 0.0001, ns: *p* > 0.05). **(I)** Stress fiber content detected by phalloidin staining.

Adhesion assays were performed on plates coated with Type I collagen (Col I), fibronectin (FN), or laminin (LN) to simulate different extracellular matrix (ECM) components. As shown in Figures 5A–G, shCTGF Vero cells exhibited a significantly reduced adhesion capacity (by approximately 50%) across all three substrates compared to control cells. In a wound healing assay, CTGF knockdown also led to a slight but consistent decrease in cell migration (Figure 5G) (Figure 5H), which may be associated with the modest, non-significant reduction in cell growth noted earlier. Furthermore, phalloidin staining revealed a marked reduction in both the abundance and structural complexity of actin stress fibers in shCTGF cells (Figure 5I).Collectively, these results indicate that CTGF knockdown reduces the adhesive capacity, migration potential, and stress fiber content of Vero cells.

### 3.6 CTGF knockdown promotes vero cell proliferation and VSV virus replication

**FIGURE 6:**
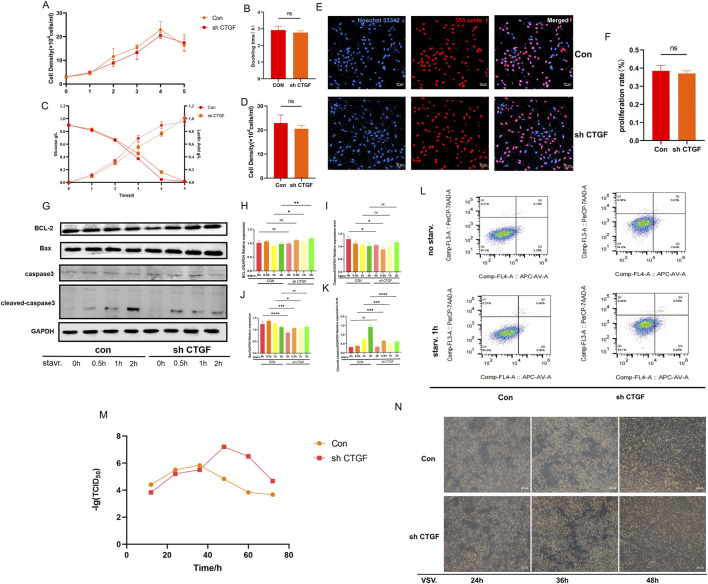
Knock down CTGF does not affect the proliferation ability, apoptosis and enhanced VSV virus replication in Vero cells. **(A)** Cell growth rate. **(B)** Doubling time. **(C)** Metabolic consumption curve showing glucose consumption and lactate production. **(D)** Maximum cell proliferation concentration. **(E)** Cell proliferation capacity detected by EdU assay and **(F)** its quantitatively calculated proliferation rate results. **(G)** Western blot analysis of BCL-2, Bax, caspase-3, and Cleaved caspase-3 expression under starvation conditions. **(H–K)** Densitometric analysis confirmed that following knockdown, the expression levels of BCL-2, Bax, and caspase-3 showed no significant differences, while Cleaved caspase-3 gradually decreased (n = 3 per group; *: *p* < 0.05, **: *p* < 0.01, ***: *p* < 0.001, ****: *p* < 0.0001, ns: *p* > 0.05). **(H)** Quantitative analysis of BCL-2. **(I)** Quantitative analysis of caspase-3. **(J)** Quantitative analysis of Bax. **(K)** Quantitative analysis of Cleaved caspase-3. **(L)** Flow cytometry detection of apoptosis under starvation stress. **(M)** LgTCID50 at different time points post-VSV infection and **(N)** Images of cytopathic effect at 24h, 36h, and 48 h.

First, we evaluated the proliferative capacity of Vero cells after CTGF knockdown.Growth curve analysis revealed a modestly shortened time in knockdown cells (25 h) compared to control cells (28 h) (Figure 6A) (Figure 6B). However, the maximum cell density was lower in knockdown cells (2 × 10^5^ cells/mL) relative to controls (2.5 × 10^5^ cells/mL), while no significant differences were observed in glucose consumption or lactate production (Figure 6C) (Figure 6D). EdU incorporation assays further confirmed no significant difference in proliferative capacity, consistent with the growth curve data (Figure 6E) (Figure 6F). Above all, these results demonstrate that CTGF knockdown does not substantially impair cell proliferation, despite a mild reduction in final cell density.

To investigate the potential role of CTGF in apoptosis regulation (Isshiki et al., 2024), we assessed starvation-induced apoptosis. Western blot analysis showed no significant differences in the expression levels of BCL-2, Bax, or full-length caspase-3 within 2 h starvation (Figure 6G) (Figure 6H) (Figure 6I (Figure 6J) (Figure 6K). However, the level of cleaved caspase-3 was significantly reduced upon CTGF knockdown, suggesting that CTGF depletion may partially alleviate starvation-induced apoptosis. Flow cytometry analysis (Figure 6L) further indicated no significant difference in the overall apoptosis rate under starvation conditions (Kim et al., 2021). Taken together, these findings indicate that CTGF knockdown moderately attenuates starvation-induced apoptosis without affecting cellular proliferation.

To determine whether CTGF knockdown affects viral susceptibility in Vero cells, we assessed their sensitivity to VSV using TCID_50_ assays. Viral growth curves showed that in both sh CTGF and control cells (Figure 6M) (Figure 6N), viral titers initially increased and then decreased between 24 and 72 h. However, from 12 to 36 h, titers in control cells were slightly higher than those in shCTGF cells. At 48 h, the viral production capacity of shCTGF cells (10^−4^·^833^/0.1 mL) was significantly higher than that of control cells (10^−7^·^2^/0.1 mL). These results indicate that CTGF knockdown significantly promotes VSV replication in Vero cells.

### 3.7 CTGF knockdown enhances the short-term suspension adaptation capacity of Vero cells

**FIGURE 7:**
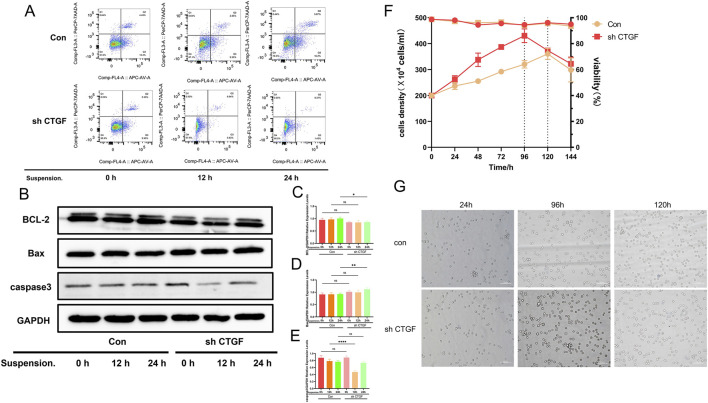
CTGF knockdown reduces apoptosis during short-term suspension adaptation and enhances the cell’s capacity for short-term suspension adaptation. **(A)** Apoptosis levels detected by flow cytometry in cells after short-term suspension adaptation at 0h, 12h, and 24 h. **(B)** Western blot analysis of BCL-2, Bax, and caspase-3 expression under short-term suspension adaptation. **(C–E)** Densitometric analysis confirmed that following knockdown, BCL-2 and caspase-3 decreased, while Bax expression increased (n = 3 per group; *: *p* < 0.05, **: *p* < 0.01, ***: *p* < 0.001, ****: *p* < 0.0001, ns: *p* > 0.05). **(C)** Quantitative analysis of BCL-2. **(D)** Quantitative analysis of Bax. **(E)** Quantitative analysis of caspase-3. **(F)** Suspension growth state and **(G)** cell morphology, under short-term suspension conditions.

To explore the effect of CTGF knockdown on apoptosis during the early stages of suspension adaptation, we assessed apoptosis at 0h, 12h, and 24 h post-adaptation using Western blot and flow cytometry (Figure 7A). The Q3 and Q2 regions represent late apoptosis and early apoptosis, respectively. Upon CTGF knockdown, the shCTGF group exhibited a late apoptosis rate of 3.33% and an early apoptosis rate of 9.56% at 0h, compared to 4.43% and 13.4% in the control group, indicating that CTGF knockdown significantly reduced the apoptosis rate at the initial time point. Notably, during early adaptation, the reduction in apoptosis upon CTGF knockdown became more pronounced. At 12h, the early and late apoptosis rates in the shCTGF group were 0.94% and 0.83%, respectively, compared to 2.85% and 9.39% in the control group. By 24h, the effect was even more significant: the shCTGF group showed late and early apoptosis rates of 0.37% and 1.43%, respectively, compared to 5.07% and 16.2% in the control group. Concurrently, Western blot analysis was performed to detect the expression levels of apoptosis-related proteins (Bcl-2, Bax, and caspase-3) (Figure 7B) (Figure 7C) (Figure 7D) (Figure 7E). Compared to the control group, the shCTGF group showed a downward trend in the expression of the anti-apoptotic protein Bcl-2, an upward trend in the expression of the pro-apoptotic protein Bax, and significantly lower caspase-3 expression at 12 h post-adaptation. Taken together, these results indicate that CTGF knockdown significantly reduces apoptosis during the early stages of suspension adaptation in Vero cells.

Subsequently, we adapted CTGF-knockdown Vero cells to suspension culture and monitored cell concentration and viability over a short-term adaptation period (0h, 24h, 48h, 72h, 96h, 120h, and 144 h) (Figure 7F). Compared to control cells, CTGF-knockdown cells exhibited enhanced adaptability to suspension growth. The adaptation period was shortened by approximately 24 h, and the shCTGF group achieved a higher maximum cell density (4.5 × 10^6^ cells/mL) compared to the control group (3.5 × 10^6^ cells/mL). Observation of morphological changes in the two suspension cell groups revealed no significant differences (Figure 7G). These results demonstrate that CTGF knockdown significantly reduces the apoptosis rate during early adaptation in Vero cells, accelerates the suspension adaptation process, and increases cell density.

## 4 Discussion

This study addressed the challenges of severe autophagy and poor suspension adaptability in Vero cell suspension culture. Moreover, knockdowning of CTGF (CCN2) significantly enhances the suspension adaptability of Vero cells by concurrently attenuating autophagic activity and cell adhesion capacity-two major bottlenecks in the industrial transition from adherent to suspension culture systems. Our findings provide both mechanistic insight and a practical genetic engineering strategy to facilitate scalable vaccine production.

The successful establishment of an EBSS-induced Vero cell autophagy model enabled transcriptomic screening under controlled stress conditions. RNA-seq analysis revealed significant upregulation of CTGF alongside other growth factors and apoptosis regulators, suggesting its potential role in stress adaptation. KEGG enrichment further highlighted pathways related to growth factor signaling and negative regulation of autophagy, supporting the notion that CTGF may function as a modulator of autophagic flux. The time-dependent accumulation of CTGF during starvation, validated at both mRNA and protein levels, reinforced its relevance in the autophagy process and positioned it as a promising candidate for genetic intervention.

Among them, the most notable is growth factor signaling also plays an important role in autophagy regulation (Chen et al., 2019). Zheng Dong et al. observed that following acute kidney injury (AKI), upregulated autophagy in renal tubular cells was accompanied by enhanced secretion of fibroblast growth factor 2 (FGF2), which contributed to renal fibrosis (Livingston et al., 2023). This indicates that cytokine expression can be upregulated under autophagic conditions. He Qiaojun et al. found that excessive autophagy in cardiac cells leads to apoptosis due to degradation of the cargo protein Toll-interacting protein (TOLLIP) and connective tissue growth factor (CCN2/CTGF), resulting in cardiomyocyte apoptosis and cardiotoxicity (Xu et al., 2022). Inhibition of high mobility group box 1 (HMGB1) prevented CCN2 degradation and provided cardioprotective effects, suggesting that growth factors can be degraded during autophagy. These results imply that growth factors play a key role in modulating autophagy, although the primary downstream receptors regulating autophagy in response to growth factors remain unclear.In this study, 21 genes with log2(Fold Change) > 1.5 were selected for transcriptomic validation via RT-qPCR. The results showed a gradually increasing trend in CTGF/CCN2 expression, which was consistent with Western blot analysis. Therefore, CTGF was selected as a candidate gene for subsequent genetic modification.

CTGF is an important transformation marker in the TME, and previous studies have shown that it mainly regulates cell adhesion (Shen et al., 2021). Previously, Shiloach et al. identified Siat7e as an adhesion-related gene in HeLa cells via DNA microarrays (Jaluria et al., 2007). Xu et al. used lentiviral transduction to generate Vero cells stably expressing TMPRSS2 and MSPL, facilitating PEDV amplification (Wang et al., 2020). To investigate the functional role of CTGF, we established a stable knock down cell line using lentiviral shRNA. Despite moderate knockdown efficiency (∼50%), which may be attributed to limited Vero genome annotation (Kiesslich and Kamen, 2020)or potential compensation by other CCN family members (Holbourn et al., 2008), significant phenotypic changes were observed. Immunofluorescence for Vimentin and CK-18 confirmed preserved epithelial-like properties, ensuring safety as non-tumorigenic cells. A stable CTGF-knockdown Vero cell line was successfully constructed, providing a candidate for suspension adaptation.The knock down of CTGF in cells exhibited reduced autophagic flux, evidenced by decreased LC3-II/I ratio, P62 accumulation, and impaired lysosomal acidification and cathepsin B activity. These findings align with studies highlighting the interplay between lysosomal function and autophagy; for instance, Kim et al. demonstrated that USF2 inhibits lysosome biogenesis by competing with TFEB (Kim et al., 2024), underscoring the importance of lysosomal activity in autophagic regulation. In addition to modulating autophagy, CTGF knockdown markedly reduced cell adhesion to extracellular matrix proteins (Col I, FN, and LN), consistent with its known role in integrin-mediated signaling and cytoskeletal organization. This approach echoes previous successful genetic interventions to improve suspension adaptation, such as Siat7e overexpression in MDCK cells (Chu et al., 2009). The observed decrease in F-actin complexity and mild reduction in migration further support CTGF’s role in cytoskeletal dynamics, although its effects in non-tumorigenic Vero cells appear less pronounced than in cancer models.

Notably, growth curves, EdU assays, and flow cytometry confirmed that CTGF knockdown did not significantly impair cell proliferation or apoptosis under starvation conditions, and even accelerated short-term suspension adaptation. This phenotypic stability is crucial for bioprocessing applications, where maintaining high cell viability and growth rates is essential. The slight reduction in maximum cell density may reflect compensatory transcriptional mechanisms (Ma et al., 2019). Under short-term suspension adaptation conditions, CTGF-knockdown Vero cells exhibited improved cell density and reduced doubling time, indicating that CTGF knockdown facilitates suspension acclimation. Compared to previously reported suspension-adapted Vero cells, our CTGF knockdown approach offers several distinct advantages. Traditional suspension adaptation methods often rely on prolonged gradual selection, which can take months and may select for undesirable traits. In contrast, our genetic engineering approach achieves enhanced suspension adaptability within weeks while maintaining critical cell characteristics. Additionally, whereas conventional adaptation often results in prolonged doubling times, CTGF-knockdown cells maintain normal proliferation rates. The reduced autophagy and adhesion in our engineered cells directly addresses the two major bottlenecks in suspension culture, potentially offering more stable and predictable bioprocess performance. This enhanced adaptability has important implications for vaccine manufacturing, as demonstrated by the approximately 200-fold increase in VSV titer at 48 h post-infection in CTGF-knockdown cells, suggesting that our engineered cells may enable higher-yield vaccine production with shorter production cycles. Future studies should examine the replication of additional vaccine-relevant viruses (e.g., rabies, polio, influenza) in these cells to fully establish their utility for vaccine production. The primary objective of this study was to investigate the mechanistic role of CTGF in short-term suspension adaptation of Vero cells, rather than to deliver a fully validated industrial production cell line. We have confirmed the short-term stability of CTGF knockdown and observed consistent phenotypic effects across multiple independent experiments. However, long-term stability is a critical requirement for industrial applications, and future studies should focus on evaluating the stability of CTGF knockdown and suspension phenotypes during prolonged culture, as well as optimizing large-scale bioprocess parameters. Our findings provide a mechanistic foundation and a candidate cell line for such subsequent industrial development. Overall, the knockdown cells not only retained sustained proliferative capacity but also demonstrated enhanced short-term suspension adaptability.

In conclusion, this study identifies CTGF as a dual regulator of autophagy and adhesion in Vero cells and demonstrates that its knockdown mitigates both processes without compromising key physiological attributes. These findings not only provide a promising engineered cell line for suspension adaptation but also contribute to a broader understanding of CTGF’s role in cellular stress response. Future work should focus on long-term suspension culture validation and viral propagation efficiency to fully assess the potential of CTGF-knockdown Vero cells in vaccine production.

## Data Availability

The datasets presented in this study can be found in online repositories. The names of the repository/repositories and accession number(s) can be found in the article/Supplementary Material.

## References

[B1] Ammerman N. C. Beier-Sexton M. Azad A. F. (2008). Growth and maintenance of vero cell lines. Curr. Protoc. Microbiol. 11, A.4E.1–A.4E.7. 10.1002/9780471729259.mca04es11 19016439 PMC2657228

[B2] Andreani N. A. Renzi S. Piovani G. Ajmone Marsan P. Bomba L. Villa R. (2017). Potential neoplastic evolution of Vero cells: *in vivo* and *in vitro* characterization. Cytotechnology 69, 741–750. 10.1007/s10616-017-0082-7 28386771 PMC5595746

[B3] Bradham D. M. Igarashi A. Potter R. L. Grotendorst G. R. (1991). Connective tissue growth factor: a cysteine-rich mitogen secreted by human vascular endothelial cells is related to the SRC-induced immediate early gene product CEF-10. J. Cell Biol. 114, 1285–1294. 10.1083/jcb.114.6.1285 1654338 PMC2289134

[B4] Chen P. S. Wang M. Y. Wu S. N. Su J. L. Hong C. C. Chuang S. E. (2007). CTGF enhances the motility of breast cancer cells *via* an integrin-alphavbeta3-ERK1/2-dependent S100A4-upregulated pathway. J. Cell Sci. 120, 2053–2065. 10.1242/jcs.03460 17550972

[B5] Chen Y. T. Chen F. W. Chang T. H. Wang T. W. Hsu T. P. Chi J. Y. (2019). Hepatoma-derived growth factor supports the antiapoptosis and profibrosis of pancreatic stellate cells. Cancer Lett. 457, 180–190. 10.1016/j.canlet.2019.05.001 31078734

[B6] Chu C. Lugovtsev V. Golding H. Betenbaugh M. Shiloach J. (2009). Conversion of MDCK cell line to suspension culture by transfecting with human siat7e gene and its application for influenza virus production. Proc. Natl. Acad. Sci. U. S. A. 106, 14802–14807. 10.1073/pnas.0905912106 19706449 PMC2728112

[B7] Holbourn K. P. Acharya K. R. Perbal B. (2008). The CCN family of proteins: structure-function relationships. Trends Biochem. Sci. 33, 461–473. 10.1016/j.tibs.2008.07.006 18789696 PMC2683937

[B8] Isshiki T. Naiel S. Vierhout M. Otsubo K. Ali P. Tsubouchi K. (2024). Therapeutic strategies to target connective tissue growth factor in fibrotic lung diseases. Pharmacol. Ther. 253, 108578. 10.1016/j.pharmthera.2023.108578 38103794

[B9] Jaluria P. Betenbaugh M. Konstantopoulos K. Frank B. Shiloach J. (2007). Application of microarrays to identify and characterize genes involved in attachment dependence in HeLa cells. Metab. Eng. 9, 241–251. 10.1016/j.ymben.2006.12.001 17240181 PMC2001267

[B10] Jiang C. G. Lv L. Liu F. R. Wang Z. N. Na D. Li F. (2013). Connective tissue growth factor is a positive regulator of epithelial-mesenchymal transition and promotes the adhesion with gastric cancer cells in human peritoneal mesothelial cells. Cytokine 61, 173–180. 10.1016/j.cyto.2012.09.013 23073116

[B11] Jun J. I. Lau L. F. (2011). Taking aim at the extracellular matrix: CCN proteins as emerging therapeutic targets. Nat. Rev. Drug Discov. 10, 945–963. 10.1038/nrd3599 22129992 PMC3663145

[B12] Kiesslich S. Kamen A. A. (2020). Vero cell upstream bioprocess development for the production of viral vectors and vaccines. Biotechnol. Adv. 44, 107608. 10.1016/j.biotechadv.2020.107608 32768520 PMC7405825

[B13] Kim B. Kim H. Jung S. Moon A. Noh D. Y. Lee Z. H. (2020). A CTGF-RUNX2-RANKL axis in breast and prostate cancer cells promotes tumor progression in bone. J. Bone Min. Res. 35, 155–166. 10.1002/jbmr.3869 31505052

[B14] Kim H. Son S. Ko Y. Shin I. (2021). CTGF regulates cell proliferation, migration, and glucose metabolism through activation of FAK signaling in triple-negative breast cancer. Oncogene 40, 2667–2681. 10.1038/s41388-021-01731-7 33692467

[B15] Kim J. Yu Y. S. Choi Y. Lee D. H. Han S. Kwon J. (2024). USF2 and TFEB compete in regulating lysosomal and autophagy genes. Nat. Commun. 15, 8334. 10.1038/s41467-024-52600-2 39333072 PMC11436898

[B16] Kular L. Pakradouni J. Kitabgi P. Laurent M. Martinerie C. (2011). The CCN family: a new class of inflammation modulators? Biochimie 93, 377–388. 10.1016/j.biochi.2010.11.010 21130134

[B17] Li M. Sun N. Peng R. Ma F. Wang J. Qiao Z. (2025). Transcriptomic analysis of suspended Vero cells and reduction of cellular autophagy by epidermal growth factor. Sheng Wu Gong Cheng Xue Bao 41, 1671–1689. 10.13345/j.cjb.240530 40328724

[B18] Litwin J. (1992). The growth of Vero cells in suspension as cell-aggregates in serum-free media. Cytotechnology 10, 169–174. 10.1007/BF00570893 1369212

[B19] Livingston M. J. Shu S. Fan Y. Li Z. Jiao Q. Yin X. M. (2023). Tubular cells produce FGF2 *via* autophagy after acute kidney injury leading to fibroblast activation and renal fibrosis. Autophagy 19, 256–277. 10.1080/15548627.2022.2072054 35491858 PMC9809951

[B20] Logan M. Rinas K. Mcconkey B. Aucoin M. G. (2022). Vero cells gain renal tubule markers in low-calcium and magnesium chemically defined media. Sci. Rep. 12, 6180. 10.1038/s41598-022-10221-z 35418617 PMC9008052

[B21] Ma Z. Zhu P. Shi H. Guo L. Zhang Q. Chen Y. (2019). PTC-bearing mRNA elicits a genetic compensation response *via* Upf3a and COMPASS components. Nature 568, 259–263. 10.1038/s41586-019-1057-y 30944473

[B22] Paillet C. Forno G. Kratje R. Etcheverrigaray M. (2009). Suspension-Vero cell cultures as a platform for viral vaccine production. Vaccine 27, 6464–6467. 10.1016/j.vaccine.2009.06.020 19559123

[B23] Ramazani Y. Knops N. Elmonem M. A. Nguyen T. Q. Arcolino F. O. VAN Den Heuvel L. (2018). Connective tissue growth factor (CTGF) from basics to clinics. Matrix Biol. 68-69, 44–66. 10.1016/j.matbio.2018.03.007 29574063

[B24] Rhim J. S. Schell K. Creasy B. Case W. (1969). Biological characteristics and viral susceptibility of an African green monkey kidney cell line (Vero). Proc. Soc. Exp. Biol. Med. 132, 670–678. 10.3181/00379727-132-34285 4982209

[B25] Rourou S. VAN DER Ark A. VAN DER Velden T. Kallel H. (2007). A microcarrier cell culture process for propagating rabies virus in Vero cells grown in a stirred bioreactor under fully animal component free conditions. Vaccine 25, 3879–3889. 10.1016/j.vaccine.2007.01.086 17307281

[B26] Rourou S. Ben Zakkour M. Kallel H. (2019). Adaptation of Vero cells to suspension growth for rabies virus production in different serum free media. Vaccine 37, 6987–6995. 10.1016/j.vaccine.2019.05.092 31201054

[B27] Schober J. M. Chen N. Grzeszkiewicz T. M. Jovanovic I. Emeson E. E. Ugarova T. P. (2002). Identification of integrin alpha(M)beta(2) as an adhesion receptor on peripheral blood monocytes for Cyr61 (CCN1) and connective tissue growth factor (CCN2): immediate-early gene products expressed in atherosclerotic lesions. Blood 99, 4457–4465. 10.1182/blood.v99.12.4457 12036876

[B28] Shen C. F. Guilbault C. Li X. Elahi S. M. Ansorge S. Kamen A. (2019). Development of suspension adapted Vero cell culture process technology for production of viral vaccines. Vaccine 37, 6996–7002. 10.1016/j.vaccine.2019.07.003 31288997

[B29] Shen Y. W. Zhou Y. D. Chen H. Z. Luan X. Zhang W. D. (2021). Targeting CTGF in cancer: an emerging therapeutic opportunity. Trends Cancer 7, 511–524. 10.1016/j.trecan.2020.12.001 33358571

[B30] Wang X. Qiao X. Sui L. Zhao H. Li F. Tang Y. D. (2020). Establishment of stable Vero cell lines expressing TMPRSS2 and MSPL: a useful tool for propagating porcine epidemic diarrhea virus in the absence of exogenous trypsin. Virulence 11, 669–685. 10.1080/21505594.2020.1770491 32471322 PMC7550007

[B31] Xu Z. Jin Y. Gao Z. Zeng Y. DU J. Yan H. (2022). Autophagic degradation of CCN2 (cellular communication network factor 2) causes cardiotoxicity of sunitinib. Autophagy 18, 1152–1173. 10.1080/15548627.2021.1965712 34432562 PMC9196717

