# Supplementary figures and images for: CTGF knockdown in Vero cells reduces autophagy and adhesion and promotes short-term suspension adaptation
Source: Front Bioeng Biotechnol. 2026 Mar 17;14:1777187. doi: 10.3389/fbioe.2026.1777187 (PMC13035795; doi:10.3389/fbioe.2026.1777187)

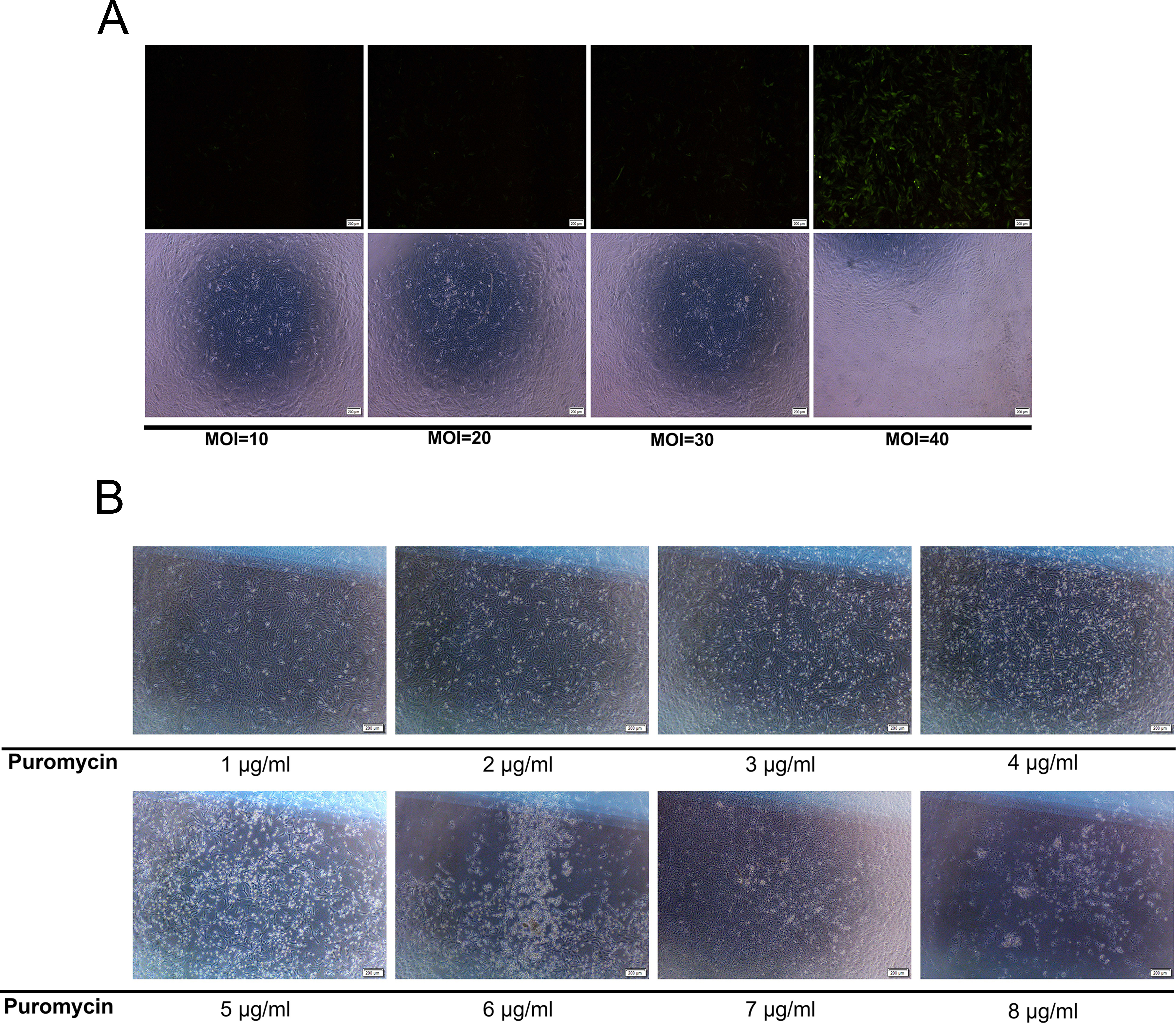

Supplement: Supplementary file 1 [file Image1.jpeg]
