# Supplementary material for: CTGF knockdown in Vero cells reduces autophagy and adhesion and promotes short-term suspension adaptation
Source: Front Bioeng Biotechnol. 2026 Mar 17;14:1777187. doi: 10.3389/fbioe.2026.1777187 (PMC13035795; doi:10.3389/fbioe.2026.1777187)

GAPDH

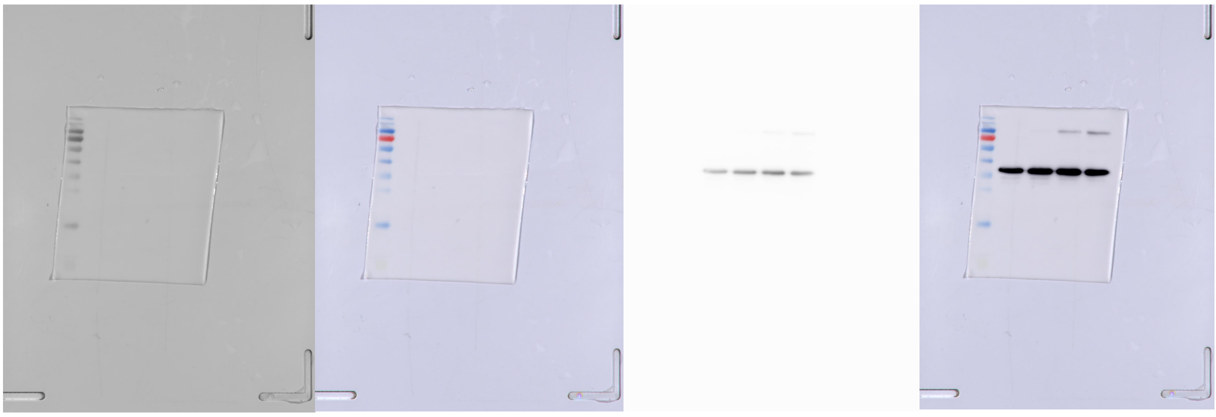

LC3

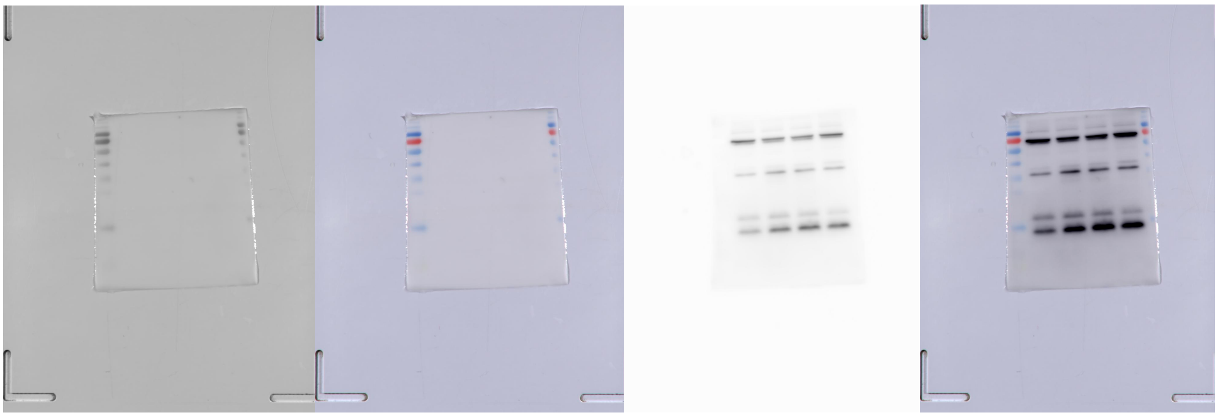

P62

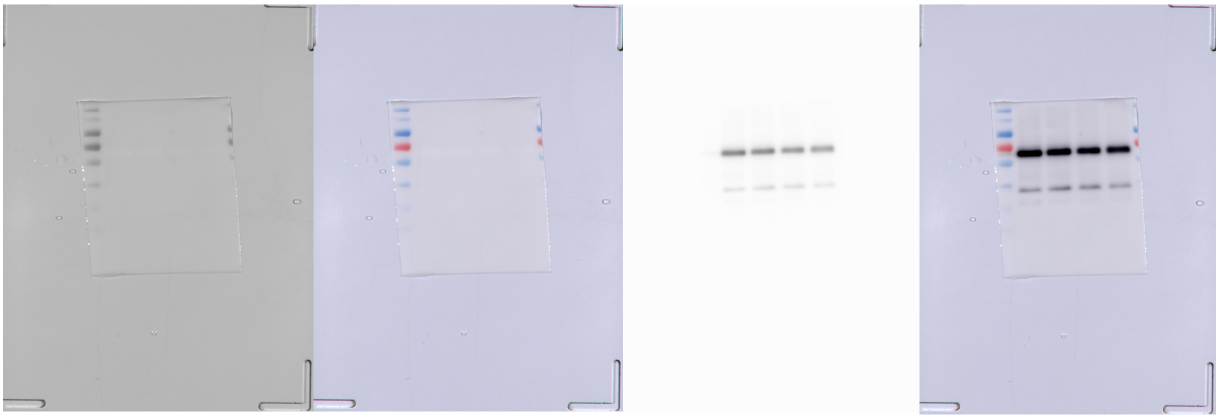

GAPDH

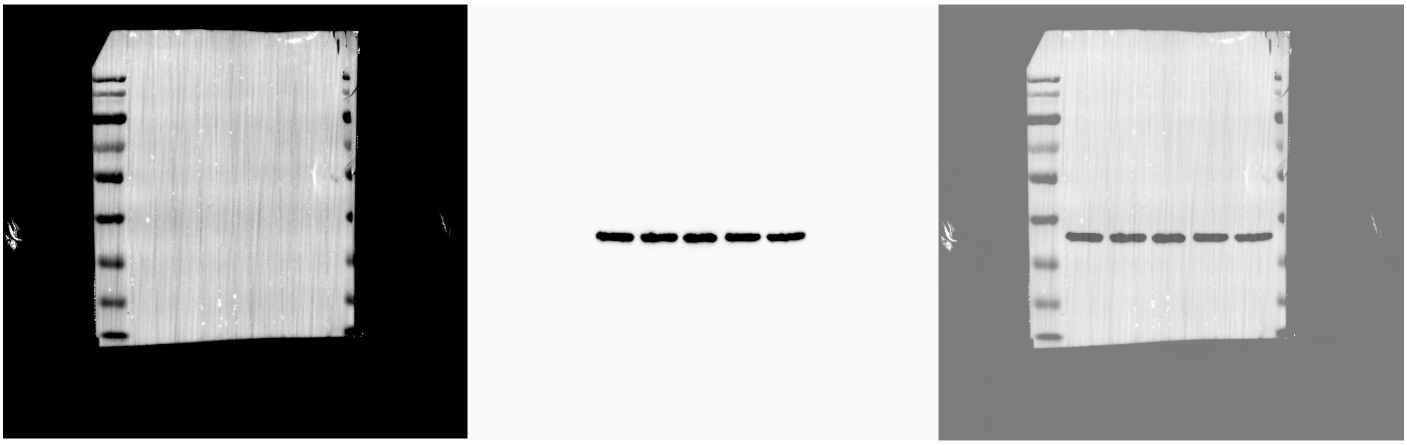

CTGF

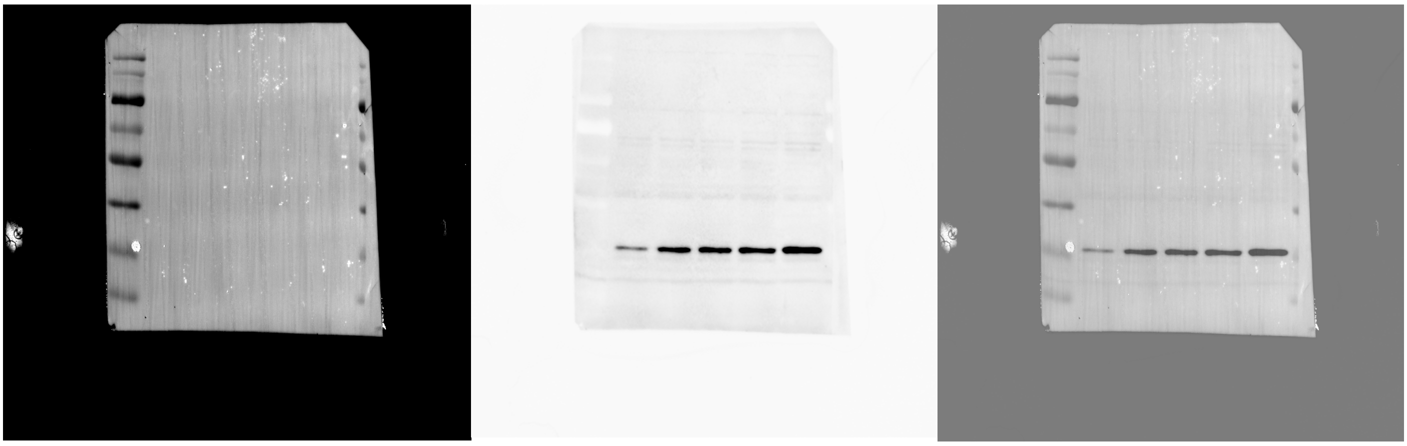

GAPDH

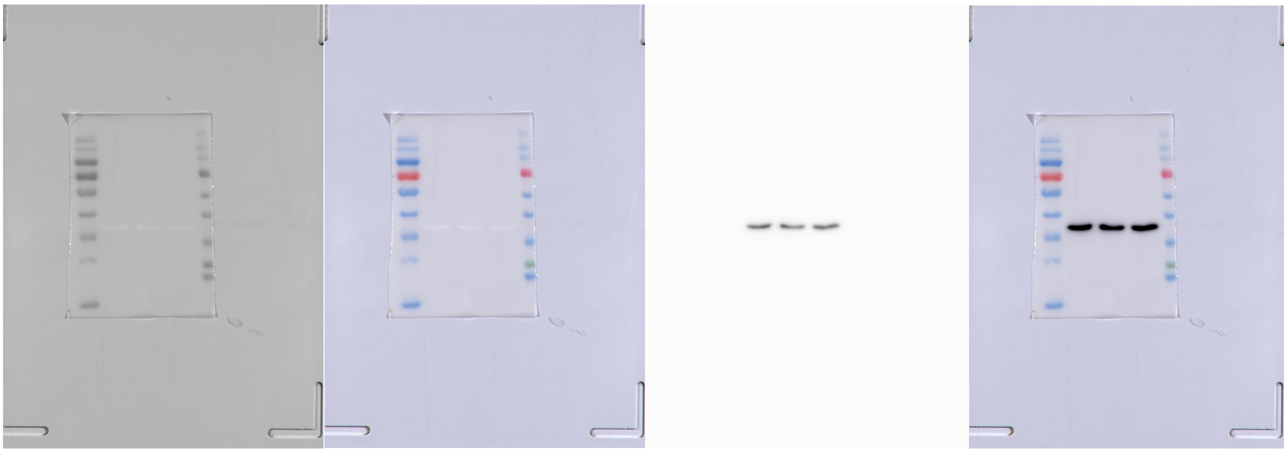

CTGF

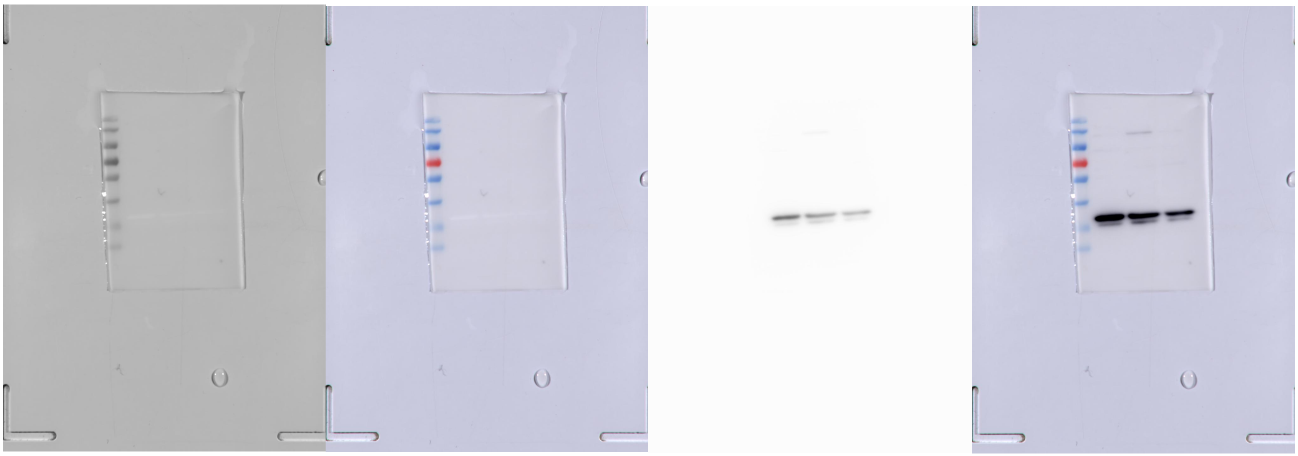

GAPDH

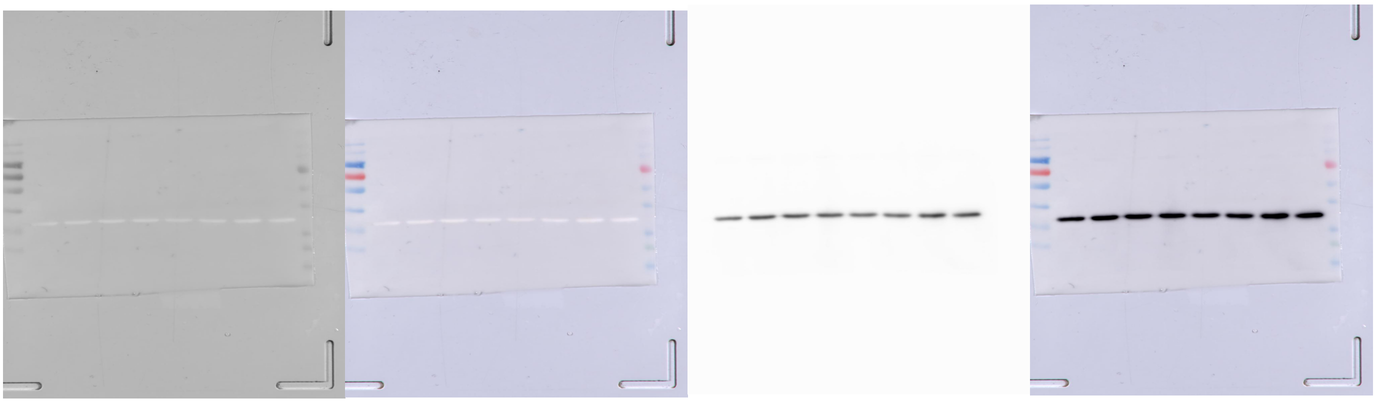

P62

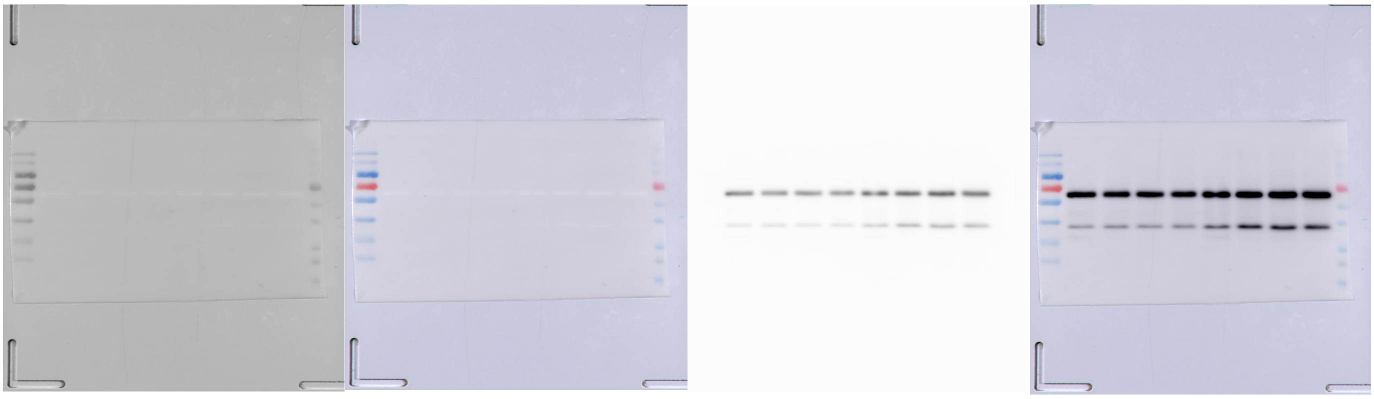

LC3

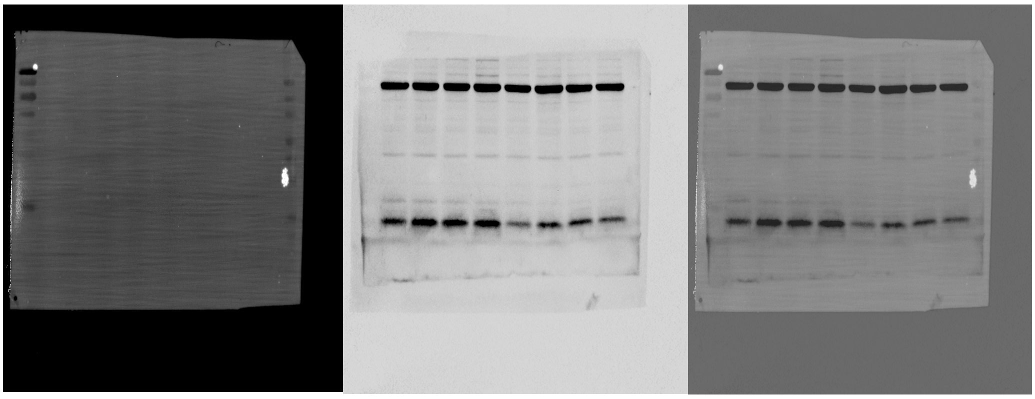

GAPDH

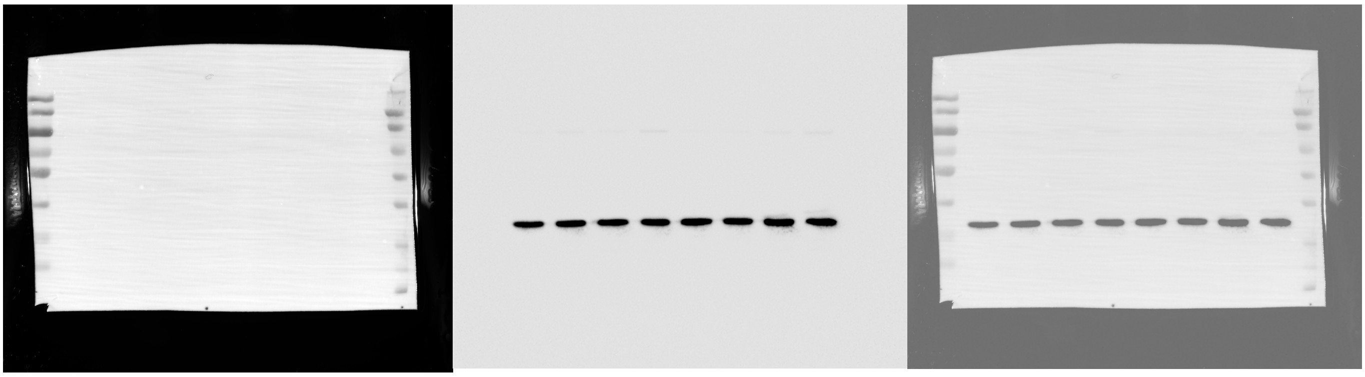

Bax

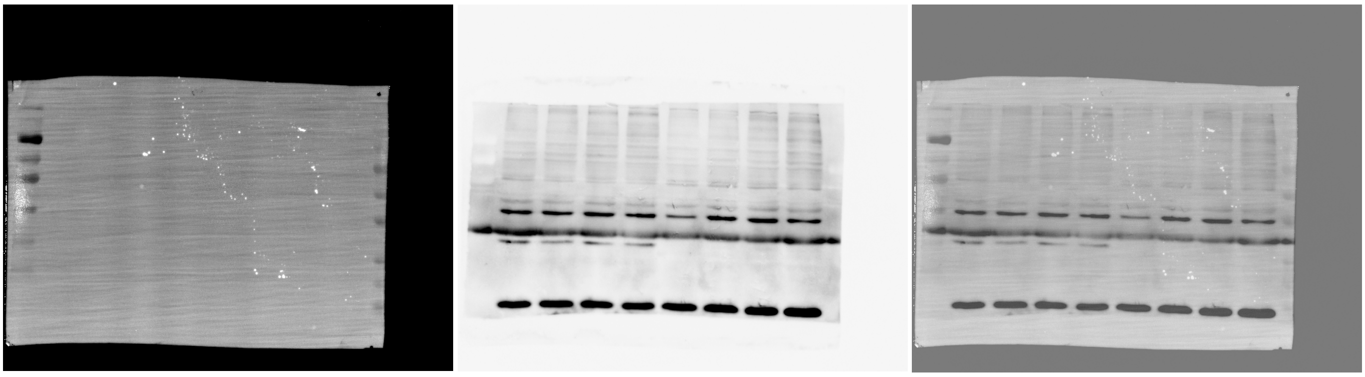

Bcl-2

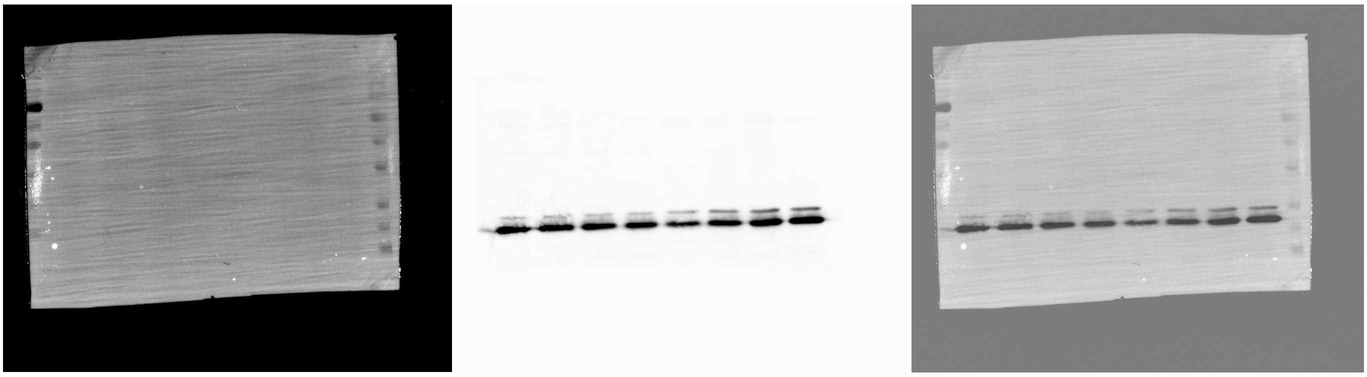

caspase3

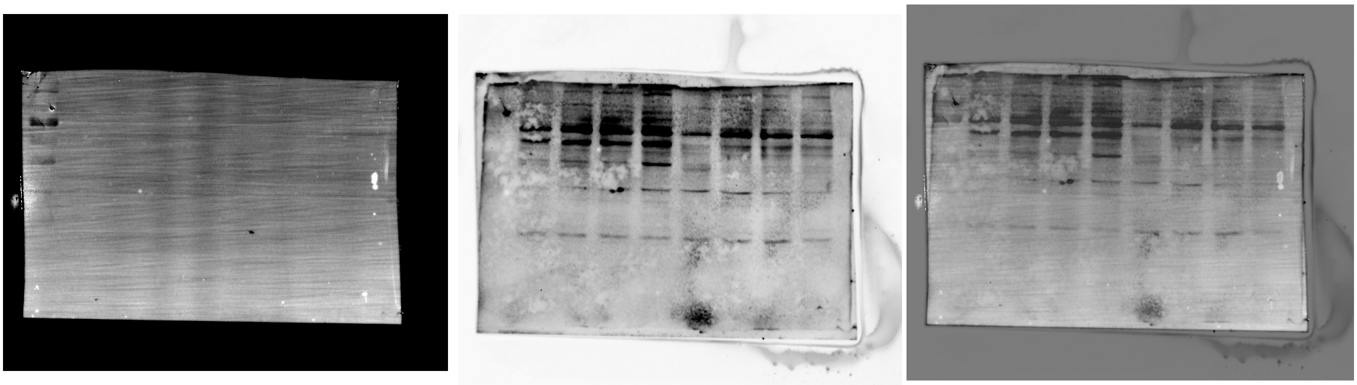

cleaved caspase3

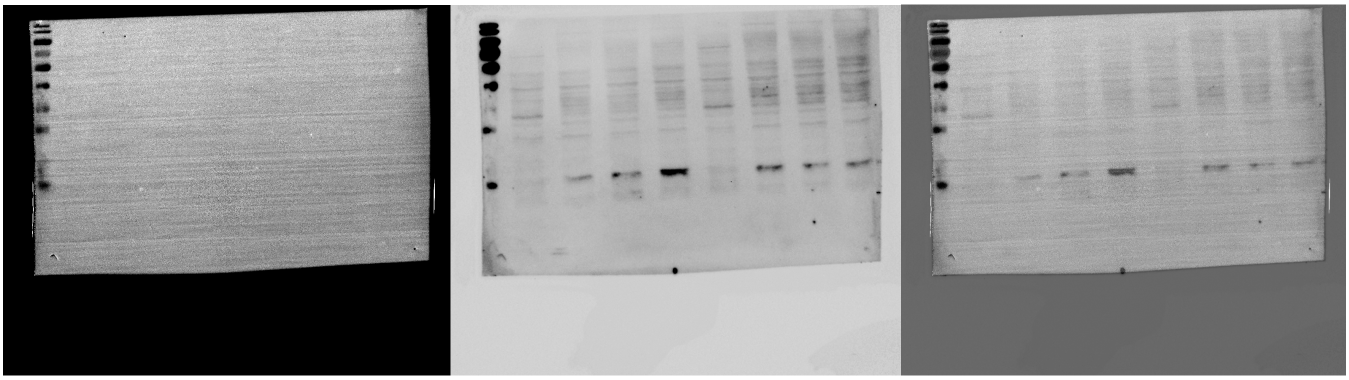

GAPDH

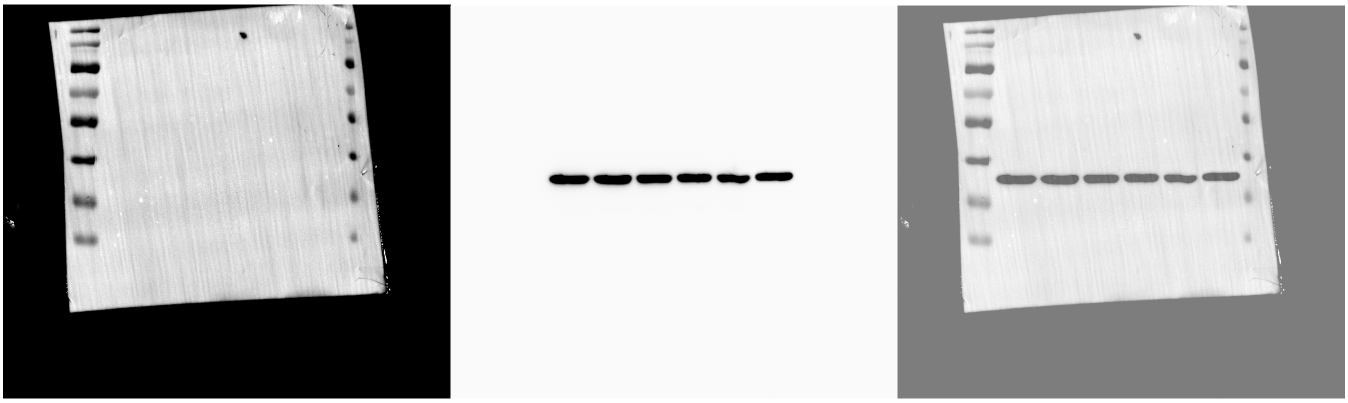

Bax

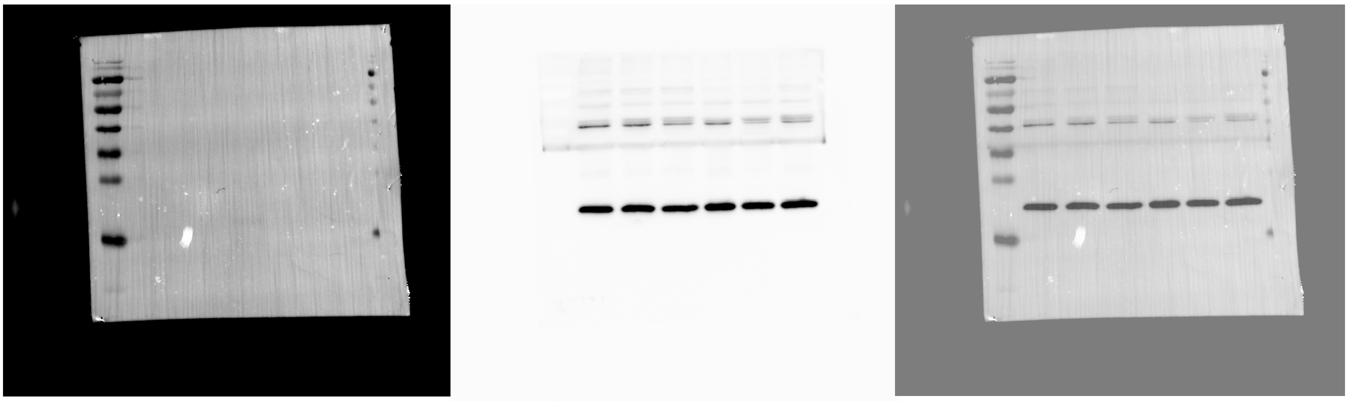

Bcl-2

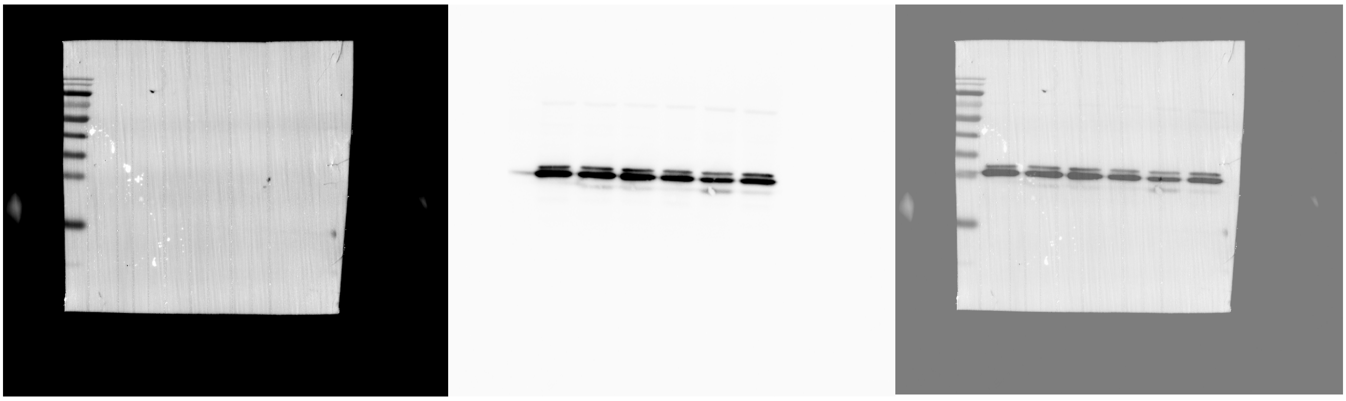

caspase3

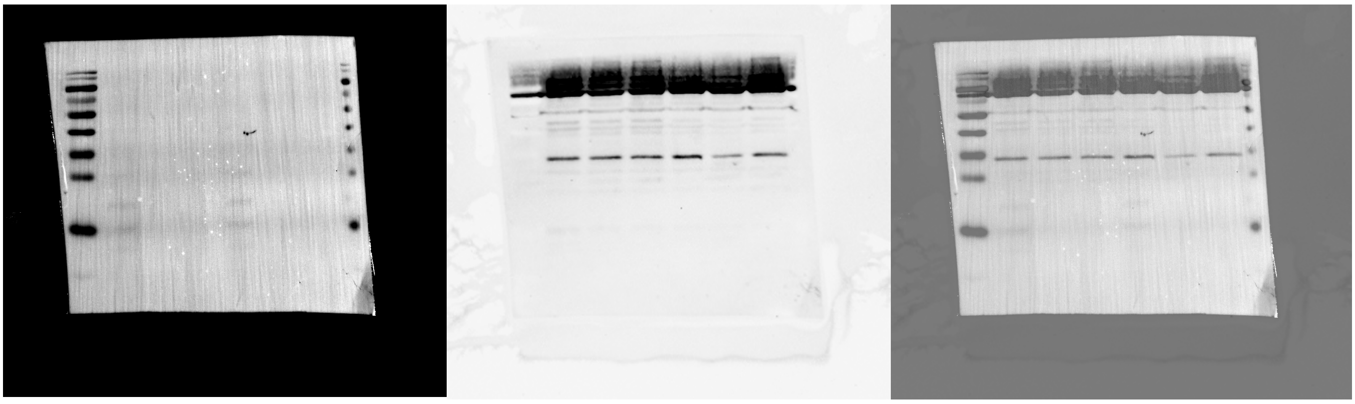

Supplement: Supplementary file 2 [file DataSheet1.pdf]
